# Children With Unilateral Cerebral Palsy Utilize More Cortical Resources for Similar Motor Output During Treadmill Gait

**DOI:** 10.3389/fnhum.2020.00036

**Published:** 2020-02-14

**Authors:** Matthew R. Short, Diane L. Damiano, Yushin Kim, Thomas C. Bulea

**Affiliations:** ^1^Functional and Applied Biomechanics Section, Rehabilitation Medicine Department, National Institutes of Health, Bethesda, MD, United States; ^2^Sports Health Rehabilitation, Cheongju University, Cheongju, South Korea

**Keywords:** electroencephalography, hemiplegia, muscle synergies, coherence, walking, pediatric, electromyography

## Abstract

Children with unilateral cerebral palsy (CP) walk independently although with an asymmetrical, more poorly coordinated pattern compared to their peers. While gait biomechanics in unilateral CP and their alteration from those without CP have been well documented, cortical mechanisms underlying gait remain inadequately understood. To the best of our knowledge, this is the first study utilizing electroencephalography (EEG) during treadmill gait in older children with and without CP. Lower limb surface electromyographic (EMG) data were collected and muscle synergy analyses performed to quantify motor output. Our primary goal was to evaluate the relationships between cortical and muscle activation within and across groups and hemispheres to provide novel insights into neural control of gait and how it may be disrupted by an early unilateral brain injury. Participants included 9 children with unilateral CP, mean age 16.0 ± 2.7 years, and 12 with typical development (TD), mean age 14.8 ± 3.0 years. EEG data were collected during a standing baseline and treadmill walking at self-selected speed. EMG of 16 lower limb muscles were also collected bilaterally and synchronized with EEG. No significant group differences were found in synergy number or structure across groups. Six cortical clusters were identified as having gait-related activation and all contained participants from both CP and TD groups; however, the percent of individuals per group appearing in different clusters varied. Notably, the cluster least represented in CP was the non-dominant motor region. Both groups showed mu-band ERD in the motor clusters during gait although sustained beta-band ERD was not evident in TD. The CP group showed greater cortical activation than TD during walking as measured by mu- and beta-ERD in the dominant and non-dominant motor and parietal regions and elevated low gamma-activity in the frontal and parietal areas, a unique finding in CP. CP showed greater bilateral motor EEG-EMG coherence in the gamma-band with the hallucis longus compared to TD. In summary, individuals with CP display increased cortical activation during gait possibly relating to differences in distal motor control of the more affected side. Strategies that iteratively reduce cortical activation while improving selective motor control are needed in CP.

## Introduction

Cerebral palsy (CP) describes a group of functional motor disabilities that are the consequences of brain injuries early in development. Movement difficulties may be predominantly unilateral (one side of the body) or bilateral (both sides), and the range of disability can vary from mild coordination problems to being totally dependent for mobility and care, as categorized by the Gross Motor Functional Classification System (GMFCS) (Palisano et al., 1997). Nearly all children with unilateral CP learn to walk independently. However, their motor patterns and coordination differ from their peers without CP with distal limb involvement most prominent (Winters et al., 1987). While gait analysis has been used extensively to describe temporal, spatial and kinematic characteristics of walking in unilateral CP, the cortical mechanisms that influence gait function in CP are not well understood and are likely to vary across and within CP subtypes, and perhaps are best characterized at the individual level (Weinstein et al., 2018).

The advancement of mobile neuroimaging technologies [e.g., functional near infrared spectroscopy (fNIRS) and electroencephalography (EEG)] and associated signal processing techniques have provided novel insights on the role of cortical activity in walking. fNIRS measures the concentration of oxygenated and de-oxygenated hemoglobin in cortical tissue, corresponding to changes in neural activity. Gait-related increases in hemodynamic activity have been reported in multiple brain regions using fNIRS, including prefrontal, premotor, primary motor and supplementary motor areas (Miyai et al., 2001; Suzuki et al., 2004). Walking tasks of greater complexity (Koenraadt et al., 2014) or requiring increased precision (Kurz et al., 2012) have been shown to further elevate hemodynamic activity.

Electroencephalography has a higher temporal resolution than hemodynamic methods such as fNIRS and therefore is commonly used to quantify movement planning and execution. Despite its low spatial resolution, high density EEG provides scalp coverage that, when combined with sophisticated processing, can resolve movement-related activations to focal scalp and/or source regions. Notably, recent EEG studies have shown that modulation of cortical activity in multiple frequency bands and originating from distinct brain regions is coupled with gait cycle phases during walking in healthy adults (Gwin et al., 2011; Severens et al., 2012; Seeber et al., 2014; Bradford et al., 2015; Bulea et al., 2015). This cortical activity is typically evaluated using relative changes in the power spectra over time, termed event-related spectral perturbations (ERSPs) (Makeig, 1993). When computed for analysis of activity within a stride, ERSPs represent differences in spectral power between a given time point in the gait cycle relative to the mean. Cortical involvement in gait can also be characterized by increases or decreases in spectral power relative to a quiet baseline (e.g., standing), termed event-related synchronization (ERS) or event-related desynchronization (ERD), respectively. Mu- (8–13 Hz) and beta- (14–30 Hz) band ERD in the motor areas of the brain are well established correlates of movement preparation and execution while beta-ERS has been associated with movement suppression or inhibition (Pfurtscheller and Da Silva, 1999; Solis-Escalante et al., 2012). During walking in adults, mu- and beta-ERD have been reported in the sensorimotor and posterior parietal regions relative to quiet standing (Severens et al., 2012; Seeber et al., 2014; Bulea et al., 2015). Mu- and beta-ERD magnitude also appear to be proportional to task difficulty as studies have found enhanced ERD in more challenging walking conditions such as those requiring active speed control (Bulea et al., 2015; Nordin et al., 2019), walking with robotic assistance (Wagner et al., 2012) and during adaptation of step length in response to perturbations (Wagner et al., 2016). Cortical modulations in other frequency bands, in particular low gamma (25–50 Hz), have also been identified during gait in prefrontal, sensorimotor and parietal areas with preliminary evidence suggesting that these rhythms may also be task-related given their modulation across different walking tasks (Wagner et al., 2012, 2014; Bulea et al., 2015; Seeber et al., 2015).

Because of the relatively low signal-to-noise ratio, EEG signals recorded from scalp electrodes during walking contain broadband contamination from movement-related artifacts (Castermans et al., 2014; Kline et al., 2015). However, studies have also shown that decomposition of EEG channels using principal component analysis applied over sliding windows (Mullen et al., 2013; Bulea et al., 2014) and independent component analysis (ICA) (Snyder et al., 2015) can parse movement artifacts from cortical activity based on their power spectra, scalp maps, dipolarity, time-frequency decompositions and lack of correlation with neighboring channels (i.e., volume conduction). The same techniques can also be used to separate electrocortical activity from physiological sources of artifact such as scalp and neck EMG, EOG, and EKG and non-physiological noise such as parasitic voltage drops from sudden skin-electrode impedance changes and electrical line noise (Makeig et al., 1996; Delorme et al., 2012). Thus, careful application of advanced signal processing techniques is necessary to ensure that the ERSPs and ERD/ERS computed from EEG collected during walking represent signal changes originating from the cortex.

While data are beginning to accumulate in healthy adults, studies that utilize mobile neuroimaging techniques to evaluate gait in typically developing children or in individuals with brain injuries are very limited, especially in children with CP. One small pilot study found that children with bilateral CP exhibited increased sensorimotor and parietal activity during walking compared to children without CP, as measured with fNIRS (Kurz et al., 2014). Perhaps relevant to gait performance, another study showed that children with bilateral CP demonstrated stronger beta-band ERD in the premotor cortex and mu-band (or alpha-band) ERD, measured via magnetoencephalography, in the anterior cingulate cortex during the motor execution phase of a knee extension task (Kurz et al., 2017). *To our knowledge, this is the first EEG study of walking in CP as well as in a healthy pediatric cohort*. In upper limb tasks, EEG-based studies have found that, compared to children with typical development, individuals with child-onset brain injury (before age 13) have reduced ERD in the affected hemisphere during wrist extension (Kukke et al., 2015), hand grasping (Weinstein et al., 2018), and reach to grasp (Inuggi et al., 2018).

Muscle activation patterns as assessed by electromyography (EMG) have long been regarded as a major source of indirect evidence of central nervous system (CNS) control. Human gait involves extensive integration of CNS commands (e.g., supraspinal and spinal circuitry) and peripheral feedback, resulting in the coordinated recruitment of multiple muscles. Recent studies in the field of motor control have posed numerous theories regarding the characterization and quantification of modular control strategies describing this recruitment (Latash et al., 2007; d’Avella et al., 2015). One widely recognized interpretation of modularity suggests that groups of muscles are recruited via *synergies* representing motor outputs organized by the CNS (Tresch et al., 2002; Ivanenko et al., 2004; d’Avella and Bizzi, 2005). Sets of muscle synergies constitute task-specific and low-dimensional decompositions of complex movements. In this way, functional behaviors that require high-level coordination and balance, such as gait, are spatiotemporally simplified, thus minimizing the issues of redundancy in muscle recruitment and kinematic degrees-of-freedom. For example, previous studies have shown that six or fewer synergies, identified through non-negative matrix factorization (NNMF) of lower-limb EMG signals, account for over 90% variance of the EMG activity associated with asymptomatic walking patterns (Ivanenko et al., 2004; Chvatal and Ting, 2012; Kim et al., 2016). Furthermore, these synergies appear to activate concurrently with one or more phases of locomotion such as forward propulsion and leg deceleration during swing.

In individuals with brain injuries, the number of synergies identified during walking (based on the aforementioned 90% variance criteria) is reduced (Clark et al., 2009; Kim et al., 2018); and a lower synergy number has been shown to correlate with greater clinical severity in individuals post-stroke (Bowden et al., 2010) and with CP (Hashiguchi et al., 2018). Additionally, synergy structures exhibited by children with CP across a bout of walking show higher variability than by those with typical development (Kim et al., 2018) while maintaining repeatable weighting and activation matrices at the individual level (Steele et al., 2019) during overground walking.

In this study, we evaluated and compared cortical and muscle activation patterns in age-matched children with unilateral CP and typical development (TD) during treadmill walking. EEG source localization was used to examine and compare group and hemispheric differences in cortical activation in multiple brain regions. Cluster analysis of identified muscle synergies, as described previously (Kim et al., 2016, 2018), was utilized for the comparison of muscle activation patterns across groups. Finally, corticomuscular coherence was performed to relate cortical and EMG data. We did not expect to find a different number of cortical sources involved in gait between groups but hypothesized that there would be differences in the magnitude, extent and location of cortical activation, particularly in the sensorimotor areas of the predominantly affected hemisphere of those with unilateral CP. We also expected group differences in the power spectra modulation within the gait cycle. Consistent with previous gait studies, we hypothesized that the CP group would exhibit fewer synergies per stride on average as well as a broader range of synergy structures compared to the TD group. Finally, we hypothesized that children with TD and CP would display mu- and beta-band desynchronization during the gait cycle overlapping with significant synergy activations and that these relationships may differ in CP. The overall goal of this project was to link cortical and peripheral mechanisms and/or output to identify potential novel targets for neurorehabilitation aimed at improving mobility in those with unilateral CP.

## Materials and Methods

### Participants

In this study, participants included 9 children with unilateral CP (7 females, 2 males; age: 16.0 ± 2.7 years) and 12 with TD (8 females, 4 males; age: 14.8 ± 3.0 years) (Table 1). In recruiting for this experiment, participants with TD were selected as age-matched controls. There were no significant differences in mean age (*p* = 0.345, independent *t*-test), height (*p* = 0.922) or weight (*p* = 0.556) between groups. Of the nine children with CP, six were GMFCS Level I and three were Level II (Table 1). This protocol was approved by the Institutional Review Board (#13-CC-0110). All participants and legal guardians provided informed assent and consent before participating, respectively.

**TABLE 1:** Participant Demographics.

	Age (yrs)	Height (cm)	Weight (kg)	Handedness	Gender	GMFCS
CP1	14	162	43.7	Right	Female	I
CP2	21	166	54.0	Left	Female	II
CP3	12	149	41.2	Right	Female	I
CP4	16	180	89.2	Left	Male	I
CP5	17	161	75.7	Right	Female	I
CP6	17	178	63.3	Right	Male	I
CP7	13	156	51.1	Left	Female	II
CP8	17	156	57.2	Left	Female	I
CP9	17	174	82.9	Left	Female	II
TD1	14	171	56.5	Right	Male	–
TD2	14	167	81.6	Right	Female	–
TD3	16	165	56.4	Right	Female	–
TD4	18	166	62.8	Right	Female	–
TD5	16	171	92.4	Right	Female	–
TD6	14	154	50.5	Right	Male	–
TD7	17	160	73.9	Right	Female	–
TD8	18	177	100.4	Right	Male	–
TD9	16	164	65.9	Right	Female	–
TD10	13	168	63.9	Right	Female	–
TD11	7	123	20.8	Right	Female	–
TD12	15	183	81.1	Right	Male	–

*Gross motor functional classification system (GMFCS).*

### Procedure and Data Collection

The data analyzed in this study were part of a larger protocol investigating cortical and muscle activation differences across different treadmill walking conditions in children with CP and TD. Prior to data collection, each participant’s preferred treadmill walking speed was determined based on average pelvic velocity during overground walking, adjusted according to their level of comfort while walking on the treadmill. Participants walked for 5 min at this self-selected speed during data collection. Prior to the walking trials, participants were instructed to stand still for 2 min to obtain a non-walking (resting) baseline.

A 64-channel, wireless, active electrode EEG system (Brain Products, Morrisville, NC, United States) was positioned on each participant’s head using the 5% 10–20 international system (Easy Cap, Germany) for electrode placement and FCz as reference. Electrode impedance was maintained below 20 kΩ throughout the experiment. EEG data were collected at 1000 Hz. EMG was recorded wirelessly (Trigno Wireless, Delsys, Boston, MA, United States) at 1000 Hz from bipolar surface electrodes positioned bilaterally on the tibialis anterior (TA), medial gastrocnemius (MG), soleus (SOL), peroneus longus (PL), rectus femoris (RF), vastus lateralis (VL), medial hamstrings (MH) and hallucis longus (HL). Kinematic data were collected using ten motion capture cameras (Vicon, Denver, CO, United States) at 100 Hz. Reflective markers were placed over anatomic locations on the pelvis and lower extremities and kinematic data collection was synchronized with both EMG and EEG recordings via manual trigger. After the experiment, motion capture data were processed offline using Visual 3D (C-Motion, Germantown, MD, United States). All other data analyses were performed using custom scripts in Matlab (Mathworks, Natick, MA, United States) in conjunction with functions from the EEGLAB v13 software (Delorme and Makeig, 2004).

### Motion Capture Analysis

Kinematic data from foot markers and force plate data were used to segment walking trials into gait cycles comprised of a dominant heel-strike (DHS) followed by a non-dominant toe-off (nDTO), a non-dominant heel-strike (nDHS), a dominant toe-off (DTO) and ending just before the next DHS. The synchronized EEG and EMG data were similarly segmented into gait cycles. After gait cycle segmentation, gait speed, cadence, stance time and step length (distance between feet at DHS and nDHS) were extracted from the kinematics and compared across groups using independent *t*-tests (alpha = 0.05, two-tailed).

### EEG Data Analysis

EEG channel data were high-pass filtered at 1 Hz (5th order Butterworth). The filtered datasets of walking and quiet standing conditions were then concatenated to create a single, merged set for each subject. Channels were removed from the merged set based on the following criteria: prolonged, flat-line periods longer than 5 s, significant noise contamination indicated by a kurtosis greater than 4 standard deviations from the mean and channels insufficiently correlated with neighboring channel activity (*r* < 0.7) (Gwin et al., 2011). Channels removed from the merged data set were also removed from each individual condition (walking and standing). An average of 61 acceptable channels were retained per subject (range: 53–64). One participant in the TD group was excluded from EEG analysis because of an excessive number of noisy channels (*n* = 36). Next, an artifact subspace reconstruction (ASR) algorithm was utilized to remove movement related artifact and improve the accuracy of subsequent independent component analysis and source localization (Mullen et al., 2013). In brief, ASR identifies time periods which contain high amplitude artifacts in EEG data by comparison with a calibration EEG dataset recorded from the same subject. Channels identified to contain artifacts within each time window are removed and reconstructed from neighboring channels using a covariance matrix computed from the calibration data. For our analysis, a variance threshold of 4 standard deviations and a sliding window of 400 ms were used to identify channels containing corrupted data. The calibration dataset was derived from the merged (standing rest and walking) set, excluding time points where the fraction of removed channels using the above criteria was greater than 0.075. After ASR, EEG data were re-referenced to a common average. Channels that were removed were interpolated prior to common average referencing, but were not included in any subsequent analysis.

An extended independent component analysis algorithm (RUNICA) was applied to the merged, ASR-cleaned datasets (Makeig et al., 1996). RUNICA is a blind source separation technique that transforms EEG channel data containing cortical and non-cortical sources into static, spatially distinct and temporally independent components (ICs). Because ASR can potentially attenuate and/or remove cortical signals of physiological relevance, only the sphering and weighting matrices produced by the RUNICA decomposition of the ASR-cleaned data were kept for further analysis (Bulea et al., 2015). The IC sphering and weighting matrices were then applied to the preprocessed, unmerged datasets associated with each subject’s treadmill walking and standing conditions. These individual datasets were subject to the same process for noisy channel removal and common average referencing as the merged dataset, but were not subject to ASR. The best fitting dipole for each IC was computed using the DIPFIT toolbox in EEGLAB with a template 3-shell boundary element head model (Oostenveld and Oostendorp, 2002). EEG channel locations for each individual were warped to match the MNI brain template (Montreal Neurological Institute, Quebec, Canada) before dipole fitting. ICs with equivalent dipole fits containing greater than 20% residual variance (RV) were rejected (Bulea et al., 2015).

For the retained ICs of each subject, walking epochs (3 s in duration) were extracted starting 1 s before DHS to ensure a complete stride in each. Non-overlapping baseline epochs (3 s in duration) were generated from the quiet standing condition. Walking and standing epochs were rejected if the IC magnitude exceeded a manually determined noise threshold of 20 μV for more than one IC at any time point. To maintain consistency between measures, the same set of epochs were retained for the EEG and raw EMG data.

Power spectral density (PSD) was computed with a Fast Fourier Transform (FFT) for each walking epoch (0–500 Hz). We then computed the time-frequency decomposition [2–50 Hz, 400 points, time-warped to the median gait event latencies across groups (Bulea et al., 2015)] with FFT for the IC walking epochs to obtain gait cycle spectrograms. ERSPs were computed by subtracting the mean spectral power (averaged across time points and strides) from the epoched walking spectrograms (Gwin et al., 2011). Gait-related ICs were identified as those which had significant power modulations within the ERSP; significance thresholds were computed for ERSPs using the *bootstat* function in EEGLAB (1000 points of surrogate data shuffled across time points and strides, alpha = 0.05, two-tailed). Scalp topographies, PSDs and time-frequency decompositions were visually inspected to confirm and remove of any remaining artifactual components from each dataset (e.g., EMG components which have high power modulations above 20 Hz and topographies located at the periphery of the head model). An average of 5 dipoles were retained (range: 3–9) for each subject. One participant in the CP group was excluded from further IC analyses as no dipoles were retained (all but 2 ICs for this subject had greater than 20% residual variance; the remaining 2 were removed based on the above criteria). To assess cortical activity relative to rest, time-frequency decompositions (2–50 Hz, 400 points) were computed for the standing epochs; standing spectrograms (averaged across time points and epochs) were subsequently subtracted from the epoched walking spectrograms to produce gait-related ERD/ERS plots (Bulea et al., 2015).

Finally, ICs from both groups (CP and TD) were pooled and clustered globally by *k*-means using parameters from ERSPs (ERSP magnitudes from 8 to 30 Hz), PSD (2–50 Hz), scalp topographies (absolute value) and dipole coordinates (Talairach space). The first ten dimensions identified by principal component analysis (PCA) were retained for each clustering measure except for the dipole coordinates (3 dimensions) (Gwin et al., 2010; Bulea et al., 2015). The resulting feature vector was further reduced to 17 principal dimensions with PCA. Previous studies have used feature vectors incorporating some combination of dipole locations, scalp projections and PSD for clustering brain ICs with *k*-means (Gwin et al., 2010; Bulea et al., 2015; Luu et al., 2017). Because dipole locations and scalp projections were expected to be more variable across subjects in the CP group, we chose to include ERSPs in the feature space to more stringently classify the cortical function of each IC. The *k*-value was set to the total number of components divided by the total number of subjects across groups, rounded up to the nearest whole number. An IC was reallocated to an outlier cluster if it was 3 or more standard deviations from its assigned cluster centroid. For *post hoc* comparisons, global clusters were split into two subclusters: one containing the ICs from CP participants and one with ICs from TD participants. Owing to the unilateral involvement of our CP cohort, IC clusters that were lateralized and symmetric about the midline were reorganized from left and right to dominant (less affected) and non-dominant (more affected) clusters based on clinical assessment by the study physician in CP and the Edinburgh Handedness Inventory (Oldfield, 1971) in TD. In this data-driven approach to clustering, it is expected that not all individuals will appear in a given cluster. Overall, this method allowed for a more functionally relevant and direct comparison of ICs across groups.

Grand mean cluster ERSPs were computed by subtracting the mean spectral power (averaged globally across all time points and strides); significance thresholds for these ERSPs were recomputed using the *bootstat* function in EEGLAB (1000 points of surrogate data shuffled across time points and strides, alpha = 0.05, two-tailed). ERD/ERS plots were averaged across strides for each IC cluster. To compare the ERSPs and ERD/ERS between groups, a non-parametric bootstrapping function, *condstat*, was implemented in EEGLAB (1000 points of surrogate data shuffled across strides, alpha = 0.05, two-tailed). Time points exhibiting significant reduction in power (suppression) in the ranges of 8 to 13 Hz (mu-band) and 14 to 30 Hz (beta-band), respectively, were also marked for each IC. Previous studies have shown that frequency bands of motor related ERD can vary by age in children (Cuevas et al., 2014), however, no differences in group level ERD results were found when individual specific mu- and beta-bands were used in our cohort.

### EMG Data Analysis

Electromyographic channel data were detrended, high-pass filtered (3rd order Butterworth, 35 Hz), full-wave rectified and low-pass filtered (3rd order Butterworth, 5 Hz) to create linear envelopes. Each EMG envelope was segmented by gait cycle (DHS to DHS), normalized by the maximum activation value per channel in each gait cycle and time-interpolated (cubic spline) to 150 points. EMG signals were linearly time-warped using the built-in EEGLAB function, *timewarp* (Delorme and Makeig, 2004) to match the EEG data and ensure gait events occurred at the same median latency across outcome measures. The resulting EMG signals were averaged across strides for each individual subject.

For each participant, muscle synergies were extracted from the pre-processed, averaged EMG data using non-negative matrix factorization (NNMF) (Lee and Seung, 1999). NNMF decomposes a set of EMG into weighting and activation matrices as described by the following equation:

$$E\,M\,G_{o}=\sum_{i=1}^{n}W_{i}\,C_{i}+e;E\,M\,G_{r}=\sum_{i=1}^{n}W_{i}\,C_{i}$$

where, *EMG*_*o*_ is the original, mean EMG matrix (muscles x time), *n* is the number of muscle synergies ranging from one to sixteen, *W* is a synergy matrix (muscles × *n*) representing weighting coefficients of individual synergies, *C* is a synergy matrix (*n* × time) representing temporal profiles of synergy activations and *e* is the residual error. Matrix multiplication of *W* and *C* results in the reconstructed EMG matrix, *EMG*_*r*_ (muscles × time). Here, a 16-synergy reconstruction is equivalent to the original set of processed EMG signals. To prevent a local minima, the NNMF procedure was performed with 100 replicates for each synergy number.

Synergy number was determined by the total variance accounted for (VAF), computed as follows:

$$V\,A\,F=1-\frac{{||E\,M\,G_{o}-E\,M\,G_{r}||}^{2}}{{||E\,M\,G_{o}-m\,E\,M\,G_{o}||}^{2}}$$

where *mEMG*_*o*_ is the channel-wise average of *EMG*_*o*_. We set a VAF threshold of 90% as in previous studies (Kim et al., 2016, 2018) and selected the lowest synergy number that satisfied this requirement. The weighting coefficients and activation profiles were normalized by the maximum channel weightings and activation values, respectively, confining the magnitude of each synergy to a range of 0 to 1.

To match similar synergies within each group, *k*-means clustering with 100 replicates was utilized with squared-Euclidean distance as the evaluative distance metric. Although previous gait studies have clustered synergies using only weighting coefficients (Steele et al., 2015; Kim et al., 2016, 2018; Shuman et al., 2016) here we added the latency of peak synergy activation during the normalized gait cycle (16 weights + 1 peak time index = 17-dimensional feature space) for clustering. The peak time index was divided by the length of the normalized time vector to ensure equal parameter weighting. Incorporating this temporal index is advantageous for correctly classifying and separating synergies which have similar weight coefficients, but differ in the time domain. Calinski-Harabasz (CH) index was used to evaluate the separation between synergies of different clusters and compactness of synergies within each cluster (squared-Euclidean distance) (Maulik and Bandyopadhyay, 2002; Cappellini et al., 2016). Clustering was repeated 100 times with *k*-values of two to the total number of synergies in each group; the *k*-value that produced the local maximum in the corresponding CH indices was identified as the optimal number. The most frequently occurring optimal cluster number and the greatest CH index across the 100 iterations was selected for further analysis. Finally, synergy cluster compactness within groups and clusters was computed using the mean intraclass correlation coefficient (ICC).

### Synergy-IC Overlap

As a preliminary comparison of brain and muscle activity, we explored the relationship between IC and synergy activity at the group level for each IC cluster. Synergy activations extracted from individuals contained in each cluster were averaged and the overlap between significant periods of cluster mean ERSP modulation and temporal synergy activation was quantified. Percentage overlap (rather than correlation coefficient) was chosen due to the different frequency content between ERSP and synergy activation signal to provide a descriptive examination of the correlation between the brain and peripheral activity. Significant ERSP modulations were defined as above. Significant periods of synergy activation were marked as regions that exceeded half of the maximum temporal profile after offset subtraction and overlap were visually compared between significant ERSP modulations and synergy activations at the group level. The proposed analysis makes no assumptions regarding the type of relationship between cortical signals and synergy activations as in previously described, regression-based studies (Pirondini et al., 2017; Pei et al., 2019) and is used only as a preliminary investigation.

### EMG-IC Coherence

Coherence between EMG channels and IC activations for the motor clusters (DM, NDM) was evaluated by computing the coherence between high-pass filtered (5th order Butterworth, 1 Hz) and rectified EMG signals and IC activations, both linearly time-warped by median gait cycle latencies. EMG-IC cross-coherence was computed using zero-padded FFT across fixed-windows (400 time points, 2–50 Hz, *newcross* in EEGLAB) and was masked for significance using *bootstat* (1000 points of surrogate data shuffled across time points and strides, alpha = 0.05). Coherence values are complex numbers and therefore can be decomposed into phase and magnitude components. Phase, in this computation, represents the time lag between input signals and can be used to determine which signal is leading/lagging relative to the gait cycle. For visualization of efferent activity, we additionally masked coherence magnitude plots to only display time points where IC activations were leading EMG signals.

## Results

### Spatiotemporal Metrics

Non-dominant limb stance time relative to the gait cycle was significantly lower in the CP group (TD: 67.2 ± 0.90%, CP: 64.2 ± 2.30%; *p* < 0.001) with no significant difference in dominant limb stance time between groups (Table 2). There were no significant differences in mean treadmill speed, normalized step length and cadence between groups (Table 2), however, mean walking speed (TD: 0.99 ± 0.11 m/s, CP: 0.89 ± 0.10 m/s; *p* = 0.053), non-dominant step length (TD: 0.30 ± 0.02, CP: 0.29 ± 0.02; *p* = 0.052), and non-dominant limb cadence (TD: 104 ± 5.74 step/min, CP: 96.1 ± 10.9 steps/min; *p* = 0.055) were all greater in TD, but failed to reach statistical threshold. Comparing across limbs in the CP group, stance time (*p* < 0.001) and cadence (*p* = 0.002) were significantly lower in the non-dominant compared to dominant limb with no difference in normalized step length.

**TABLE 2:** Spatiotemporal metrics during treadmill walking.

	Preferred treadmill speed(m/s)	Normalized step length^a^		Stance time (%)		Cadence (steps/min)		
				
		Non-dominant	Dominant	Non-dominant	Dominant	Non-dominant	Dominant	Average
TD	0.99 ± 0.11	0.30 ± 0.02	0.31 ± 0.01	67.2 ± 0.90	67.4 ± 0.82	104 ± 5.74	104 ± 4.92	104 ± 5.17
CP	0.89 ± 0.10	0.29 ± 0.02	0.30 ± 0.03	64.2 ± 2.30	68.4 ± 1.61	96.1 ± 10.9	108 ± 10.8	102 ± 10.1
*p*	0.053	0.052	0.539	**<0.001**	0.072	0.055	0.287	0.598

*Mean ± standard deviation; Bold values indicate significant differences between groups (*p* < 0.05, independent *t*-test) ^*a*^Step length was normalized by participant height in meters.*

### EEG Component Clusters

**FIGURE 1:**
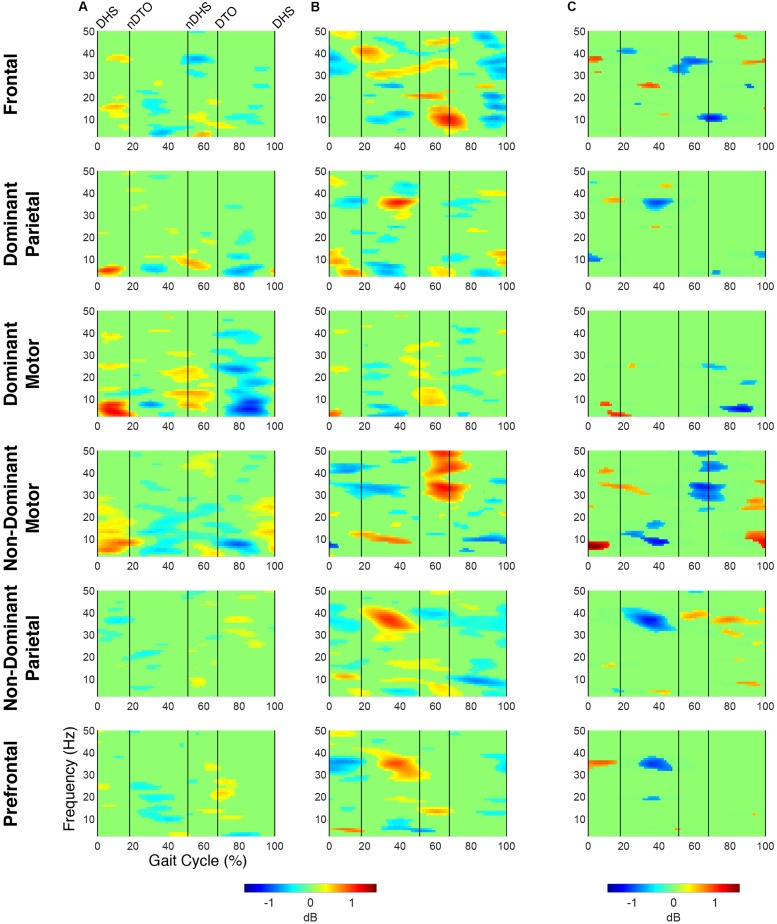
Time-frequency modulations relative to mean gait cycle activity during treadmill walking. Grand mean gait event related spectral perturbations (ERSPs) computed for each cortical cluster in **(A)** TD and **(B)** CP, displayed in dB. **(C)** Between group differences of spectrograms calculated by subtracting grand mean ERSPs in CP from TD, displayed in dB. ERSPs and difference spectrograms were masked for significance (alpha <0.05); non-significant values were set to 0 dB (green).

At the group level, global *k*-means clustering resulted in six IC clusters for preferred speed walking (2 outlier components). Clustered scalp topographies and grand mean ERSPs (Figure 1) were spatially determined to represent activity from the frontal (FR), dominant parietal (DP), dominant motor (DM), non-dominant motor (NDM), non-dominant parietal (NDP) and prefrontal (PF) regions based on the cluster centroid dipole locations in the MNI template. Brodmann areas were identified in a ±2 mm range of all individual dipoles within a given cluster (Table 3; Lancaster et al., 2000). Each of these clusters contained subsets of individuals that were considered to be representative of each group. With regard to group representation, the percentages of total subjects (respective of each group) contained in each IC cluster were most different between groups in the NDM cluster (91% of subjects with TD; 38% of subjects with CP) with differences also observed in the DP cluster (82% of subjects with TD; 56% of subjects with CP), the DM cluster (55% of subjects with TD; 75% of subjects with CP), and the PF cluster (36% of subjects with TD; 75% of subjects with CP).

**TABLE 3:** IC Cluster characteristics.

IC Clusterlocation	Brodmannareas^a^	Scalp topographies^b^		# of Subjects (ICs)		% of total subjects	
				
		TD	CP	TD	CP	TD	CP
Frontal	6, 8, 32			5 (6)	3 (5)	45%	38%
		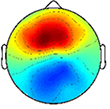	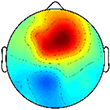				

Dominant parietal	5, 7, 18, 19, 31, 39, 40			9 (10)	5 (7)	82%	56%
		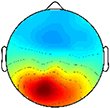	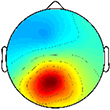				

Dominant motor	3, 4, 6, 8, 9, 22			6 (9)	6 (7)	55%	75%
		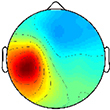	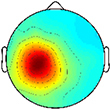				

Non-dominant motor	3, 4, 6, 8, 22, 24			10 (12)	3 (3)	91%	38%
		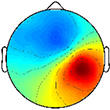	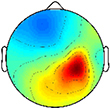				

Non-dominant parietal	5, 7, 13, 18, 19, 22, 31, 39, 40			7 (13)	6 (12)	64%	75%
		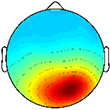	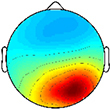				

Prefrontal	6, 8, 9, 10, 24, 32			4 (6)	6 (8)	36%	75%
		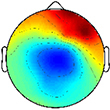	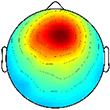				

*^*a*^Brodmann Areas were found within a ±2 mm area of all individual dipoles in each cluster ^*b*^Individual scalp topographies were inverted to best match the cluster polarity; individual topographies of the motor and parietal clusters were mirrored about the *y*-axis according to hemisphere dominance.*

At the group level, instances of mu- and beta-suppression most often occurred during single limb stance and/or swing, evidenced by the grand mean ERSPs (Figure 1). In the TD group, the DM and NDM clusters exhibited a consistent decrease in power for the duration of single stance and swing, respectively, across both the mu- and beta-bands. Low gamma-band power (25–50 Hz), in phase with mu- and beta-band modulations, also decreased in the TD motor clusters. In the CP group, the DM and NDM clusters showed periods of decreased power in the same phases of the gait cycle (single stance and swing); however, these instances were spectrally discontinuous across the mu- and beta-bands after significance masking. Additionally, gamma-band power in the DM cluster was in phase with beta-band power during early swing but appeared offset from mu- and beta-band modulations in the NDM cluster, exhibiting significant activity instead from initial contact through mid-stance.

Statistical comparison of ERSPs between groups showed significant differences in mu- and beta-band power in the DP, NDM, and FR clusters (Figure 1). In the DP cluster, the CP group had significantly more mu-band power centered around initial contact. In the NDM cluster, the CP group briefly displayed increased power in the mu-band at the beginning of single stance and decreased power in the same frequency range during loading response and terminal swing. While, descriptively, the CP group exhibited more mu-band power before and after toe-off in the FR cluster, differences in the mu- and beta-bands were not largely apparent. Significant changes in gamma-band power were observed between groups for all clusters except the DM cluster. In the DP, NDP, and PF clusters, the CP group consistently showed more gamma-band power during mid-stance. Similarly, the CP group had increased gamma-band power during double stance in the FR and NDM clusters.

The percentage of the gait cycle with mu-suppression relative to the mean was significantly greater in the TD group for the DM cluster (*p* < 0.001) (Table 4). For the NDM cluster, the percentage of mu-suppression was also greater in the TD group, however, this trend was not significant (*p* = 0.075). Conversely, the percentage of mu-suppression for the NDP cluster was greater, but not significantly, in the CP group (*p* = 0.055). No other significant differences were found when comparing mu- and beta-suppression between groups in the remaining IC clusters.

**TABLE 4:** Mu- and Beta-suppression percentage relative to gait cycle.

IC clusterlocation	Mu-suppression (%)			Beta-suppression (%)		
		
	TD	CP	*p*	TD	CP	*p*
Frontal	25.3 ± 11.5	32.9 ± 6.78	0.228	43.4 ± 10.8	46.3 ± 13.4	0.707
Dominant parietal	22.9 ± 8.20	25.9 ± 14.5	0.597	32.1 ± 12.6	39.0 ± 6.17	0.209
Dominant motor	**42.8 ± 12.7**	**17.0 ± 8.42**	**<0.001**	48.1 ± 10.8	44.7 ± 16.2	0.621
Non-dominant motor	38.6 ± 16.2	18.9 ± 13.2	0.075	44.3 ± 15.5	45.8 ± 16.1	0.884
Non-dominant parietal	17.5 ± 13.9	27.8 ± 11.3	0.055	38.3 ± 13.3	44.8 ± 9.68	0.175
Prefrontal	19.8 ± 13.9	30.3 ± 8.99	0.109	39.8 ± 14.8	35.3 ± 16.8	0.616

*Mean ± standard deviation; Bold values indicate significant differences between groups (*p* < 0.05, independent *t*-test).*

**FIGURE 2:**
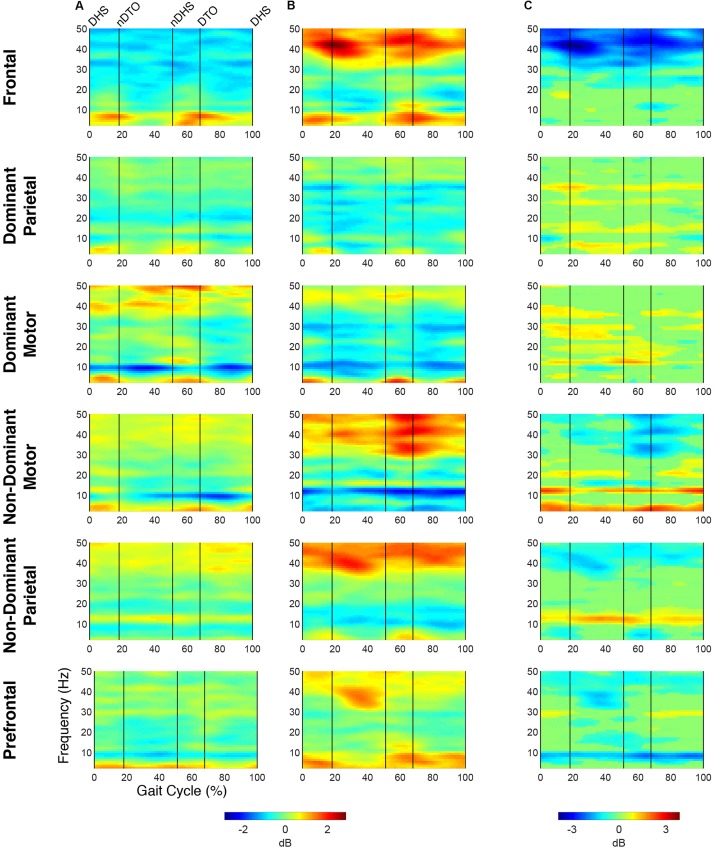
Time-frequency activity relative to quiet standing during treadmill walking. Grand mean event-related desynchronization (ERD)/event-related synchronization (ERS) plots for each cortical cluster in **(A)** TD and **(B)** CP, displayed in dB. **(C)** Between group differences of spectrograms calculated by subtracting grand mean ERD/ERS plots in CP from TD, displayed in dB. Difference spectrograms were masked for significance (alpha <0.05); non-significant values were set to 0 dB (green).

Spectrograms computed relative to quiet standing (i.e., ERD/ERS) revealed continuous desynchronization in the mu- and beta-bands across groups and clusters (Figure 2). In both groups, the strongest instances of mu-band desynchronization were present from mid-stance to initial contact in the NDM clusters and from swing to late stance in the DM clusters. In the TD group, increased gamma-band power was observed throughout the gait cycle in the DM, NDM, NDP, and PF clusters. The same clusters exhibited increased gamma-band activity in the CP group, with the addition of the FR cluster.

When comparing ERD/ERS plots between groups (Figure 2), upper mu-ERD was significantly greater for the CP group in all motor and parietal clusters with significant differences persisting throughout the gait cycle. These differences were also present in various segments of the beta-band for the DP, DM, and NDM clusters. Despite predominant instances of greater ERD in the motor clusters of the CP group, we also found phasic periods of less ERD in the CP group. These phenomena occurred in the lower mu-band during single stance and swing for the DM cluster and during terminal swing and loading response for the DP cluster as well in the lower beta-band during single and double stance for the NDM cluster. Similarly, lower beta-ERD was significantly decreased before and after toe-off in the CP group for the FR cluster. In the PF cluster, upper mu- and beta-ERD were both lower for the CP group during single and double stance.

The CP group exhibited significantly greater gamma-band power throughout the gait cycle in all IC clusters excluding those from the dominant hemisphere (DP and DM). Interestingly, the TD group showed greater gamma-band power, particularly during single stance, in the DP and DM clusters. Delta- (2–4 Hz) and theta- (4–7 Hz) band differences varied between clusters. While power was significantly decreased throughout the gait cycle in the CP group for the DP and NDM clusters, power increased during all phases of the gait cycle except single stance in the CP group for the NDP cluster at these frequencies.

### EMG Synergy Clusters

For all subjects and conditions, 4 to 6 synergies were extracted from the averaged strides using the 90% VAF criteria. The mean extracted synergy numbers for unrestricted walking in TD and CP were 5.0 ± 0.4 (12 subjects; VAF = 0.92 ± 0.02) and 5.0 ± 0.5 (9 subjects; VAF = 0.93 ± 0.02), respectively, with no significant differences discerned between groups (synergy number: *p* = 1.0; VAF: *p* = 0.74).

**FIGURE 3:**
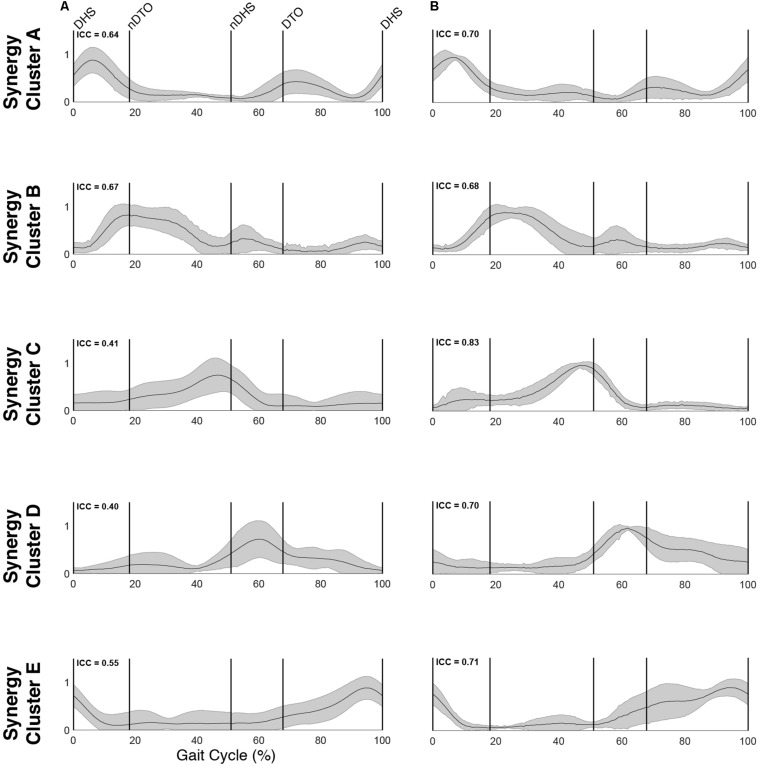
Muscle synergy activations. Mean (standard deviation) activation profiles for each synergy cluster in **(A)** TD and **(B)** CP. Mean intraclass correlation coefficients (ICC) are reported in the upper left corner of each plot.

**FIGURE 4:**
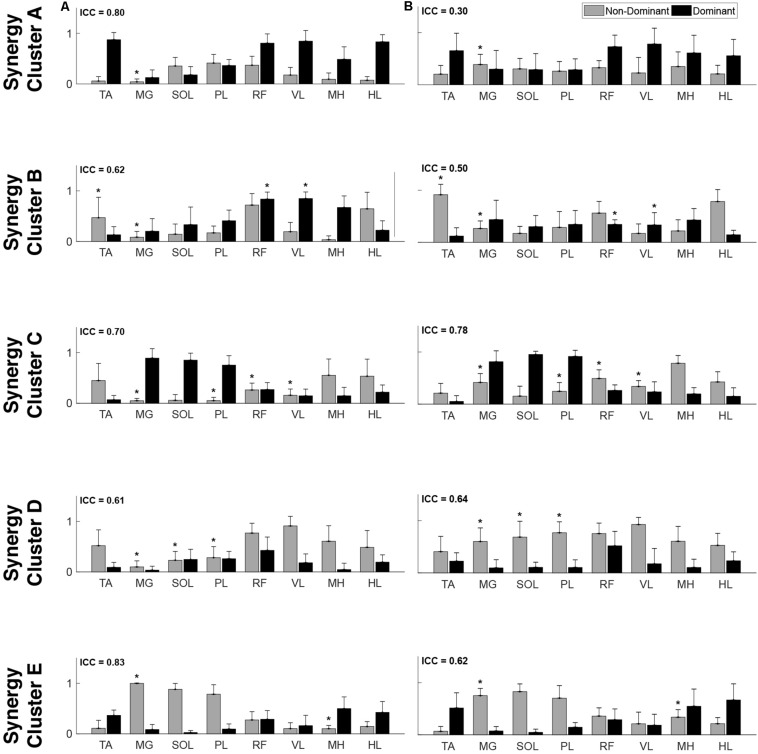
Muscle synergy weightings. Mean (standard deviation) weight coefficients for each synergy cluster in **(A)** TD and **(B)** CP. Asterisks (*) indicate significant differences between groups for individual muscle weightings, relative to each synergy cluster (*p* < 0.01, independent *t*-test). Mean intraclass correlation coefficients (ICC) are reported in the upper left corner of each plot.

The optimal synergy cluster number from *k*-means was five for both the TD and CP group (Figures 3, 4). These five clusters were ordered by the peak timing of their activation profiles and determined to be similar across groups in terms of mean activation profiles (Figures 3A,B) and weight coefficients (Figures 4A,B) (Activation Profiles: *r* = 0.95 ± 0.02; Weight Coefficients: *r* = 0.77 ± 0.16). Descriptions of the synergy clusters (referred to as Synergy Cluster A, B, C, D and E for each group) are provided below and in Table 5. With regard to cluster compactness, the TD group generally had higher average ICC values computed across weight coefficients, but lower ICC values across activation profiles compared to the CP group.

**TABLE 5:** Synergy structure characteristics.

Synergy cluster	# of Subjects (Synergies)		Activation profiles			Weight coefficients		
			
			ICC		*r*	ICC		*r*
						
	TD	CP	TD	CP	Between groups	TD	CP	Between groups
A	11 (11)	9 (10)	0.64	0.70	0.94	0.80	0.30	0.86
B	8 (8)	9 (10)	0.67	0.68	0.94	0.62	0.50	0.52
C	12 (15)	9 (9)	0.41	0.83	0.98	0.70	0.78	0.86
D	12 (14)	8 (8)	0.40	0.70	0.92	0.61	0.64	0.70
E	12 (12)	8 (8)	0.55	0.71	0.94	0.83	0.62	0.92

**r* indicates Pearson correlation coefficient computed between mean structures for each cluster.*

Cluster A, active primarily during terminal swing and loading response, promoted knee extension and foot stabilization. In the TD group, this cluster was associated with dominant TA, RF, VL, MH, and HL activity as well as non-dominant SOL, PL, and RF activity. In the CP group, this cluster exhibited similar muscle activity, with the addition of increased non-dominant MG activity. Notably, in the CP group, this cluster exhibited the lowest average ICC calculated across weight coefficients (ICC = 0.30).

Cluster B was active from loading response through mid-stance and was primarily responsible for hip extension and knee stabilization for forward progression. In the TD group, this cluster involved dominant RF, VL and MH activity as well as non-dominant TA, RF and HL activity. In the CP group, dominant RF and VL (two primary knee extensors) activity were diminished while non-dominant TA and MG activity increased.

Cluster C, active primarily during terminal stance, accounted for hip and knee extension throughout the stance phase and ankle plantarflexion in preparation of toe-off. In the TD group, this cluster was associated with dominant MG, SOL, and PL activity as well as non-dominant TA, MH, and HL activity. In the CP group, muscle activity was similar with the exception of increased non-dominant MG, PL, RF, and VL activity.

Cluster D acts as a reciprocal to cluster B, promoting support and stabilization during terminal swing and initial contact of the contralateral leg. Muscle activity was similar between groups with the exception of increased non-dominant MG, SOL, and PL activity in the CP cluster.

Cluster E acts reciprocally to cluster C, maintaining extension and initiating leg lift during mid- to terminal stance of the contralateral leg. Muscle activity was again similar between groups with the exception of increased non-dominant MG, activity in the TD group and increased non-dominant MH activity in the CP group.

### Synergy-IC Overlap

**FIGURE 5:**
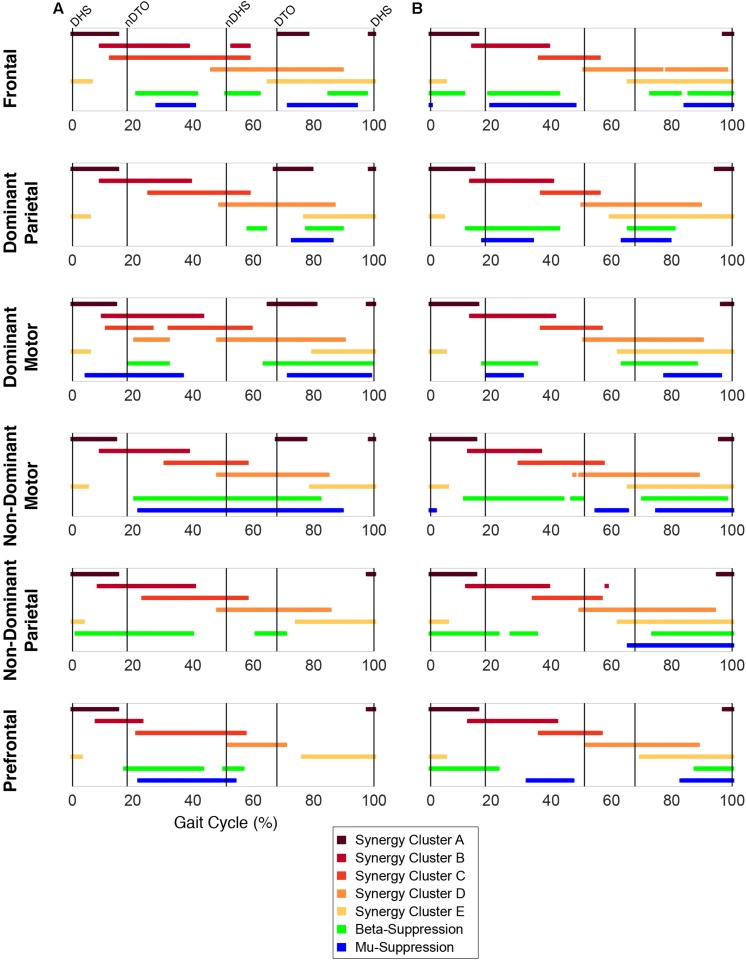
Overlap between synergy and cortical component activity. Significant periods of synergy activation plotted with mu- and beta-suppression throughout the gait cycle for each cortical cluster in **(A)** TD and **(B)** CP.

Plotting of significant synergy and IC activations across the gait cycle revealed no clear pattern of correlation between the two signals (Figure 5). The activation profiles of synergy clusters A-E were averaged relative to the subset of subjects contained in each IC cluster. Despite this reorganization, mean activation profiles were relatively consistent with grand mean results from Figures 3, 4. Synergy activation was distributed across strides, with each of the five synergy clusters locked to a particular phase of the gait cycle. Mu- and beta-suppression, as previously described, were typically coupled and occurred during multiple phases of the gait cycle, primarily during single stance and swing. Therefore IC activity overlapped with many synergies, but showed no preference in terms of timing to one particular synergy, regardless of IC cluster location or group. Most often, mu- and beta-suppression overlapped with synergy clusters B, C, and E, with the onset of suppression leading the onset of muscle activation in many cases.

### EMG-IC Coherence

**FIGURE 6:**
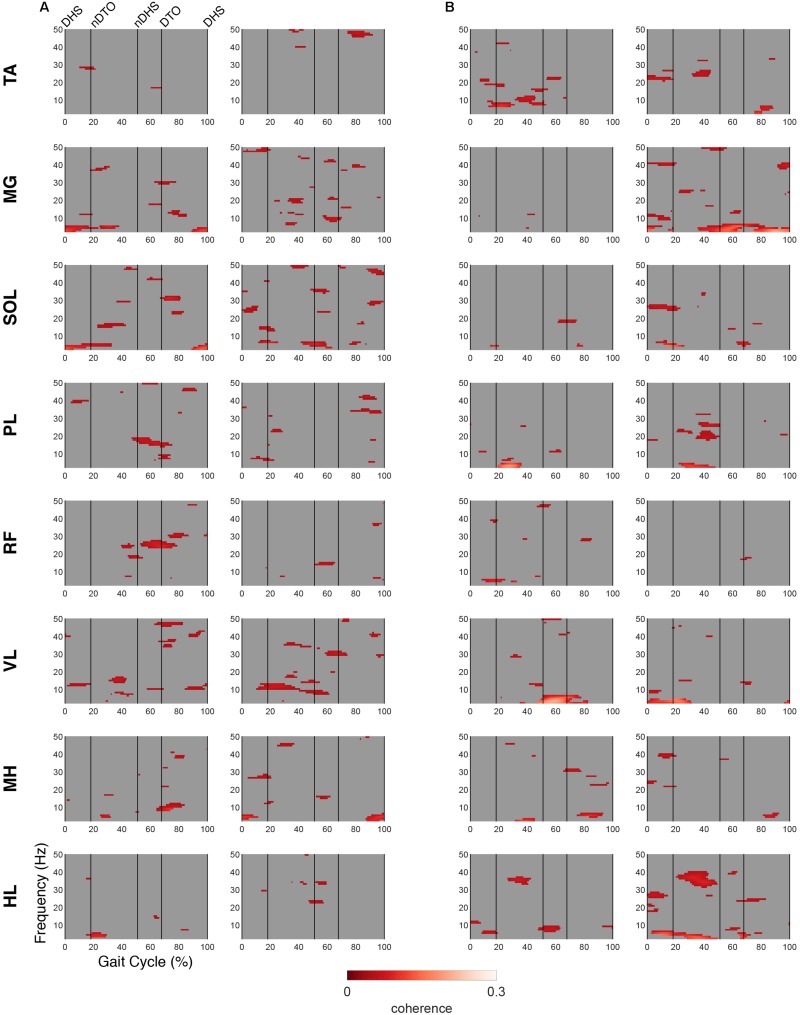
EMG-IC coherence relative to dominant motor clusters. Linear coherence magnitudes between non-dominant (left)/dominant (right) EMG signals and dominant motor IC activations in **(A)** TD and **(B)** CP. Coherence plots were masked for significance (alpha <0.05); non-significant values and time points where EMG signals led IC activations (phase >0 radians) were set to 0 (gray).

**FIGURE 7:**
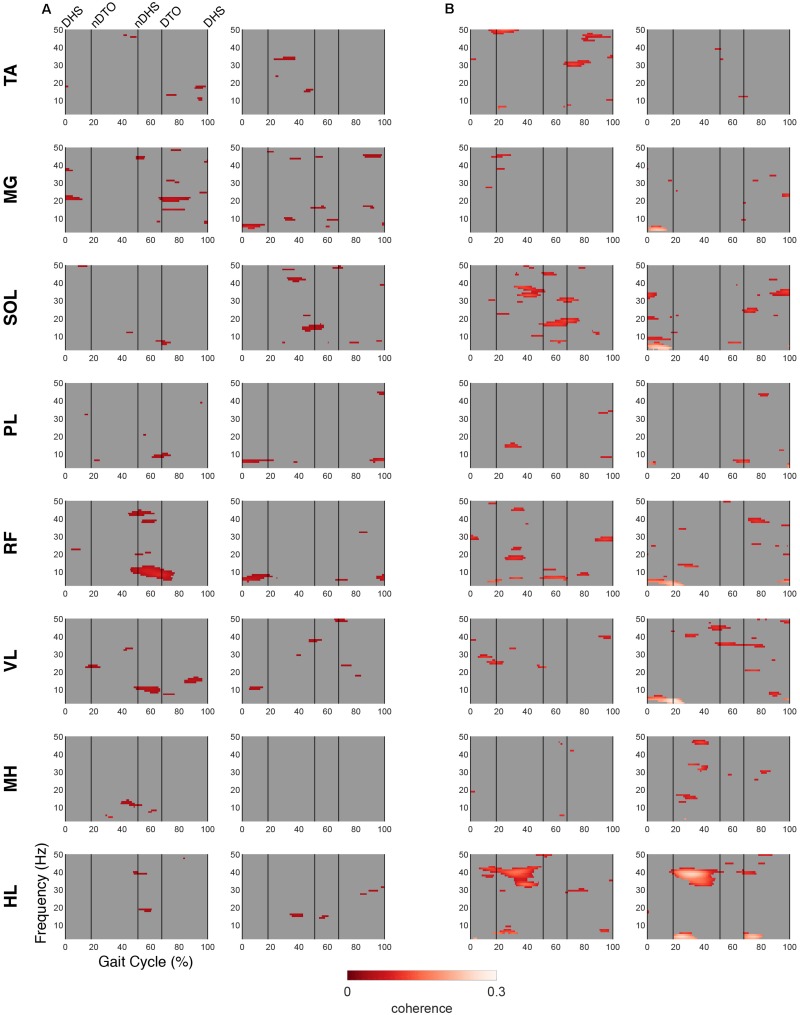
EMG-IC coherence relative to non-dominant motor clusters. Linear coherence magnitudes between non-dominant (left)/dominant (right) EMG signals and non-dominant motor IC activations in **(A)** TD and **(B)** CP. Coherence plots were masked for significance (alpha <0.05); non-significant values and time points where EMG signals led IC activations (phase >0 radians) were set to 0 (gray).

For both groups, significant periods of coherence were found across many frequencies between EMG channels and IC activations (Figures 6, 7). In the DM cluster of the TD group (Figure 6), delta-band coherence was observed in the non-dominant MG and SOL during initial double stance. Similarly, in the DM cluster of the CP group, delta-band coherence appeared in the dominant MG and non-dominant VL during terminal double stance. The CP group additionally showed gamma-band coherence bilaterally in the HL during single stance. Mu- and beta-coherence were present in the DM cluster of both groups, though these instances were scattered and not consistent across muscles.

In the NDM cluster (Figure 7), the TD group displayed less coherence compared to the DM cluster, however, mu-coherence was observed briefly during terminal double stance in the dominant RF and VL. In the NDM cluster of the CP group, delta-coherence was present during initial double stance in the dominant MG, SOL, RF, and VL. The strongest gamma-coherence in this cluster occurred during single stance, appearing bilaterally in the HL and, to a lesser extent, unilaterally in the dominant PL. These instances of gamma-coherence were similar to those found in the DM cluster in terms of timing and frequency.

## Discussion

This study represents the first evaluation of cortical activity using EEG during walking in two pediatric cohorts, one with TD and one with CP. The evaluation was performed on a treadmill rather than overground for logistical reasons, mainly to minimize motion artifact and maximize the number of strides for EEG analyses. It is important to note that the participants in the CP group were at the highest levels of functional mobility in CP and were minimally, but not significantly, slower in comfortable gait speed and in cadence on the more involved side than those with TD. Consequently, the groups showed many similarities, particularly in the synergy analyses, however, some potentially important differences were also identified. In addition, both groups, while under 21 years of age, were comprised mainly of adolescents whose gait patterns are likely to be highly similar to those of adults.

With respect to the EMG data, consistent with previous studies, between 4 to 6 synergies extracted via NNMF were able to sufficiently recreate individual channel EMG during normal walking (Ivanenko et al., 2004; Chvatal and Ting, 2012; Kim et al., 2016). However, in contrast with other findings (Steele et al., 2015; Kim et al., 2018), no significant differences were found in mean synergy numbers and VAF between individuals with CP and TD across conditions with an average of 5 synergies identified for each group. This inconsistency could be attributed in part to the high level of functioning in the CP cohort as well the number of muscles used in the synergy extraction (Kim et al., 2016) and the procedure of averaging the EMG data across strides. Comparing synergy clusters between CP and TD, activation and weight matrices were highly correlated for paired clusters. Overall, though correlated between groups, the synergy weight coefficients of the CP clusters exemplified much more non-dominant (affected) limb activity, evidenced especially in Synergy Cluster C (Figure 4). The observed differences in weight coefficients can be attributed to abnormalities in selective motor control seen in children with CP (Leonard et al., 1991; Crenna, 1998), supported by previous synergy studies in this population (Kim et al., 2018).

A previous cohort study demonstrated that children with unilateral and bilateral CP exhibited a combination of similar and disparate synergies relative to those of children with TD on a stride-to-stride basis (Kim et al., 2018). Our observation of similar synergy numbers between groups after averaging can be interpreted to represent the most frequently occurring synergies of each group. While averaging and concatenating strides has been shown to exclude relevant stride-to-stride variability of muscle activity (Oliveira et al., 2014), this was deemed a necessary step in our methodology. EEG time-frequency analysis requires a relatively large number of trials to make meaningful conclusions. Consequently, in order to directly compare synergy results with cortical activity, this stride averaging procedure was utilized consistently for both the EMG and EEG datasets.

Similar to the finding of the same number of muscle synergies in each group, the IC clustering results showed six distinct clusters that all contained cortical sources from both CP and TD participants. At the group level, we observed roughly the same peripheral output (no extraneous, voluntary movements in CP and no significant difference in gait speed) but consistent differences in cortical activation between groups within each cluster. These differences may reflect altered cognitive and/or motor requirements for execution of the same task. These results are also similar to the results found in healthy adults using nearly identical methods (Bulea et al., 2015). However, the distribution of individuals across clusters revealed some important group and hemispheric differences. Fewer subjects with CP were found to have ICs in the non-dominant motor cluster; this cluster was represented by the lowest percentage of CP across all clusters. The highest percentages of individuals with CP were in the DM and PF clusters, both of which had appreciably lower percentages in TD. These results suggest an under-reliance on the NDM region and an over-reliance on the PF and DM regions in CP, which is not surprising based on upper limb studies that demonstrate a reorganization that favors use of the dominant hemisphere over the non-dominant one in both unilateral non-dominant side and bilateral tasks (Kukke et al., 2015; Inuggi et al., 2018; Weinstein et al., 2018). These results may also be attributed to the elevated functional role of the dominant limb during walking in our cohort, as evidenced by increased dominant limb stance time and cadence compared to the non-dominant side. While the representation of individuals within each cluster varied in both groups, the results for TD were more consistent than for CP, as shown by the high percentage (91%) of those with TD represented in the NDM cluster as one example. This result is similar to earlier findings from Weinstein et al. (2018), demonstrating that each child with CP likely has their own neural signature on how their brain develops in response to early injury. Neurorehabilitation strategies that demonstrate effectiveness in shifting the reliance more toward the NDM or in lowering the PF activation during tasks that involve the more affected side warrant further exploration and development.

In line with previous work from healthy adults (Gwin et al., 2011; Severens et al., 2012; Seeber et al., 2014; Bulea et al., 2015), we observed, *for the first time* in a younger cohort, cortical activity modulated relative to the gait cycle in mu-, beta- and low gamma-bands. The motor (dominant and non-dominant) regions in the TD group had stronger within stride modulations of mu- and beta-band activity and slightly different timing patterns but were generally quite similar to CP. In the low gamma-band, the CP group had significantly greater modulations in all regions except for the dominant motor cluster. This suggests more cortical activation during gait in brain regions beyond the affected sensory and motor areas in CP. Compared to standing, the CP group displayed a greater increase in low gamma-band activity than the TD group in the frontal areas, also suggesting increased cortical resources attending to the walking task. These findings are similar to results showing elevated frontal activity in more demanding walking tasks in adults (Bulea et al., 2015; Seeber et al., 2015; Wagner et al., 2016). The frontal cortex has been implicated in elevated top-down or executive control of motor tasks (Miller and Cohen, 2001; Danielmeier et al., 2011) and thus our results suggest that children with unilateral CP dedicate more executive control to the treadmill walking task than TD. Interestingly, low gamma-modulation is also elevated in the non-dominant (more affected) motor and parietal areas of CP compared to TD. Given previous studies indicating that greater sensorimotor gamma activity is linked to tasks requiring greater dynamic control (Mehrkanoon et al., 2014), this suggests that walking is also more challenging for children with CP.

When evaluating mu-band ERD, we found that both groups showed significant desynchronization, or elevated cortical activity, in walking compared to standing as had been shown previously in healthy adults. The TD group here, however, differed from earlier results in adults (Bulea et al., 2015; Seeber et al., 2015) in that, compared to standing, strong beta-ERD in the motor and parietal areas was not present. In general, the CP group showed greater cortical activations than TD during walking as measured by mu- and beta-ERD in the NDM, DM, NDP, and DP areas. These results are interesting because they are in disagreement with some upper extremity EEG studies that show *less* task-related ERD in the motor areas. However, fNIRS results from our group (Sukal-Moulton et al., 2018) also show that children with bilateral CP display more widespread motor cortex activation than those with TD for bilateral lower extremity tasks. Greater cortical activation was associated with greater muscle activation in our earlier study, suggesting that brain effort reflects peripheral effort. We found here that, whereas the overall synergy number did not differ between groups during walking, there was increased non-dominant limb activity across multiple synergy clusters in the CP cohort (Figure 4). Enhanced mu- and beta-ERD, particularly in motor areas, is indicative of elevated sensorimotor activation during walking (Pfurtscheller and Da Silva, 1999; Severens et al., 2012; Seeber et al., 2014; Bulea et al., 2015). Interestingly, recent results also show mu- and beta-ERD in parietal areas when comparing across walking tasks of varying difficulty [e.g., active vs. passive walking (Wagner et al., 2012; Bulea et al., 2015), fast vs. slow walking (Bulea et al., 2015; Nordin et al., 2019), and step shortening adaptations (Wagner et al., 2016)]. Collectively, these results suggest that children with CP require enhanced cortical output to achieve a similar motor output as those with TD.

The primary goal of this study was to relate cortical and muscle activity during a complex bilateral task. Characterization of this relationship can be explored through simultaneous evaluation of cortical activity and synergy output. This concept has been demonstrated in a previous study using multivariate regression to model the influence of EEG frequency-band power on kinematic synergies during hand grasping (Pei et al., 2019). Another study found significant similarities between EEG microstates and muscle synergies via canonical correlation during hand reaching and grasping (Pirondini et al., 2017). To our knowledge, comparative analyses incorporating EEG and muscle synergies have not been applied to ambulation. However, at this level of analysis, we failed to find significant correlations between synergies and activation in cortical sources. The cortical motor sources presented here were active throughout the gait cycle with some relative fluctuations at specific phases, differing slightly across groups. Given that ICs represent coherent activity of large groups (i.e., thousands or more) of neurons, it is perhaps not surprising that we did not find significant associations between ICs and synergy activations.

It is expected that for a task such as gait, which coordinates subcortical and spinal pathways to move the entire body while ensuring dynamic stability and forward progression, mapping cortical to peripheral output would be far more difficult, if not impossible. However, EMG–EEG coherence studies have identified significant relationships that tend to be stronger and more consistent in static tasks isolated to a few joints, requiring higher force or effort levels (Mima and Hallett, 1999). As a next step, we performed coherence analyses relating the motor sources to EMG activation of individual muscles, focusing on the efferent control where the cortical activity would presumably lead the muscle activity. Similar patterns of delta-band coherence were found across groups for the DM region. Coherence in this frequency range persisted for the NDM region in the CP group but was not apparent in TD. In both motor regions, the CP group uniquely showed gamma-band coherence for the HL, primarily on the dominant limb, a distal muscle predominantly affected in unilateral CP, with some evidence that the dominant side may try to overcompensate to maintain optimal mobility (Wiley and Damiano, 1998).

One limitation of this study is the loss of stride-to-stride variability via gait cycle averaging as well as intra-subject variability due to group-level analysis. Averaging spectrograms at the group-level has the potential to obscure subject-specific evidence of cortical contributions to muscle recruitment. This issue is more consequential in the CP group due to the distinctive nature of each individual’s brain injury and the subsequent reorganization of cortical processes. Of particular note is the inclusion of only three children with CP in the NDM cluster. To this effect, we clustered brain ICs with an equivalent focus on spatial and functional organization using dipole locations and time-frequency parameters. However, the chance of inaccurately grouping these ICs still persists. Therefore applying the same clustering analysis at the individual level may prove more effective in characterizing relationships between the CNS and periphery, which is particularly important for clinical applications where the rehabilitation program should be tailored to the individual. Regarding EMG processing, differences in specific parameters for time-interpolation, normalization and filtering can affect the results of synergy extraction by NNMF (Shuman et al., 2017) and should be considered when comparing synergy results across studies. Finally, the group results here are based on a relatively small number of subjects, especially when comparing ICs within clusters and therefore, we did not control for multiple comparisons when looking at ERSP and ERD/ERS difference plots. Also, the cohort with CP was mildly affected with unilateral involvement and thus, these results warrant further investigation in larger samples and in different CP subtypes.

## Conclusion

Electrocortical measurements and muscle synergy analysis are independently, potentially powerful tools for neurorehabilitation to better understand and address motor control abnormalities that impact daily functional activities. However, a quantitative understanding of how motor control strategies are encoded by the CNS and communicated to the periphery is generally lacking. In this study, we compared a subtype of CP with the highest mobility levels to a group with typical development. Therefore, finding that muscle synergy weights and activations were not significantly different at the group level is not unreasonable. Still, we were able to detect unique differences in distribution of individuals across brain regions active during gait as well as significant differences that reflect the unilateral injury that primarily disrupts distal control and its cortical representation in the sensorimotor brain regions in CP. Based on our results, we advocate for the development and implementation of strategies for CP that are more personalized and which iteratively reduce cortical activation while improving selective motor control using brain-computer interface (BCI) methodologies similar to studies in stroke.

## Data Availability Statement

The datasets generated for this study are available on request to the corresponding author.

## Ethics Statement

The studies involving human participants were reviewed and approved by the Combined Neuroscience Institutional Review Board, National Institutes of Health. Written informed consent to participate in this study was provided by the participants’ legal guardian.

## Author Contributions

TB, YK, and DD conceived and designed the study. MS performed the data analysis, interpreted the data, wrote the manuscript, and created the figures. DD supervised the clinical aspects of the study, interpreted the data, and wrote the manuscript. YK collected and analyzed the data and reviewed the manuscript. TB collected the data, supervised the data analysis, interpreted the data, wrote the manuscript, and created the figures.

## Conflict of Interest

The authors declare that the research was conducted in the absence of any commercial or financial relationships that could be construed as a potential conflict of interest.
